# Impact of Training Settings on Hormonal Status, Body Weight, and Glucose Levels in Wistar Rats

**DOI:** 10.1155/jnme/8668755

**Published:** 2026-11-02

**Authors:** H. Shakoor, C. Platat, A. Riabinin, A. Parveen, J. Kizhakkayil, B. Sadek, C. Dsouza, E. Adeghate, Y. Statsenko

**Affiliations:** ^1^ Department of Nutrition and Health College of Medicine and Health Sciences United Arab Emirates University Al Ain 15551 UAE uaeu.ac.ae; ^2^ Department of Nutrition and Exercise Physiology Elson S. Floyd College of Medicine Washington State University Spokane Washington 99202 USA wsu.edu; ^3^ Department of Radiology College of Medicine and Health Sciences United Arab Emirates University Al Ain 15551 UAE uaeu.ac.ae; ^4^ Department of Pharmacology and Therapeutics College of Medicine and Health Sciences United Arab Emirates University Al Ain 15551 UAE uaeu.ac.ae; ^5^ Department of Anatomy College of Medicine and Health Sciences United Arab Emirates University Al Ain 15551 UAE uaeu.ac.ae

**Keywords:** blood glucose, body weight, hormones, machine learning, muscle secretions, physical training

## Abstract

This study explored how different physical training settings affect hormonal and metabolic responses in male Wistar rats (*n* = 28). Rats were randomly assigned to a control (no‐exercise), ET4 (4‐week training), ET8 (8‐week training), DT4 (4 week training and then 4‐week detraining), and PCA (4‐week training and then 4‐week detraining + PCA). Serum hormones, muscle secretions, glycogen, pyruvate, HK, and PFK were analyzed. The groups were compared using one‐way ANOVA, with Cohen’s d and Pearson’s correlation for associations. Machine learning models predicted body weight and glucose levels from training settings. Eight‐week training significantly elevated IL‐6 (*p* = 0.021, *d* = 2.04), IGF‐1 (*p* = 0.012, *d* = 1.96), cortisol (*p* = 0.0277, *d* = 1.677) and testosterone‐to‐cortisol (*p* = 0.009, *d* = 2.465), and leptin (*p* = 0.001, *d* = 3.098) vs. control. Furthermore, 4–8 weeks of training resulted in a significant increase in the number of skeletal muscle satellite cells compared with the respective control groups. Myostatin was reduced with 4‐week training (*p* = 0.002, *d* = 2.273) and further after 8 week (*p* = 0.014, *d* = 1.613). PCA supplementation increased pyruvate levels (*p* = 0.005, *d* = 1.31). Key correlations were found between IL‐6 and ghrelin (*r* = −0.52, *p* = 0.005), FGF‐21 and T/A (*r* = 0.47, *p* = 0.012), and IGF‐1 with aldosterone (*r* = 0.47, *p* = 0.012) and cortisol (*r* = −0.50, *p* = 0.006). Machine learning revealed total training weeks as the strongest predictor of body weight (XGBoost), while recent training (last 2 weeks) best predicted glucose levels (LightGBM). Metabolic adaptations emerged within 2 weeks, including improvement in IL‐6, IGF‐1, ghrelin, cortisol, aldosterone, and blood glucose, with effects amplified by 8 weeks of training. PCA supplementation regulated energy metabolism during detraining.

## 1. Introduction

The effect of physical exercise on body weight has been extensively studied for years. Research showed that sufficient physical activity levels can significantly improve body weight and fatness [1]. However, studies did not suggest the effective physical activity dose that would reduce health‐related risks [2]. Even though researchers focus on the association of training load with muscular power and dynamic athletic performance [3, 4], the optimal training design should establish a proper protein and energy metabolism. This served as the motivation for the current study: we tried to find the training settings that may reduce or maintain the body weight [5].

A highly individualized training process is the major challenge of studies on physical activity. Besides, intraindividual differences in eating habits, sleep patterns, and leisure activities account for the high variability in study results. Animal models help overcome human study limitations [6]. Still, genetic polymorphism and epigenetic modifications may lead to discrepant findings: biochemical shifts and physiological responses to physical activities vary among individuals [6]. To deal with this challenge, one should consider hormonal findings reflecting early metabolic changes before they become apparent at the systemic level [6].

Physical training plays a crucial role in regulating hormone levels. Metabolic and hormonal responses to exercise depend on its duration and intensity. For example, physical load enhances insulin sensitivity in short‐term observations [7] and improves the functioning of the pancreatic *β* ‐cell in the long term [8]. Training mode also impacts the level of steroids (e.g., testosterone and cortisol) [9]. Testosterone promotes protein anabolism and reduces fasting blood glucose [10]. Contrarily, the secretion of cortisol stimulates protein catabolism and elevates fasting blood glucose due to the inhibition of *β*‐cell activity [11]. Hence, one can improve glucose metabolism by regulating steroid synthesis through sports participation.

Other hormonal responses to physical training may also alter glucose homeostasis. For instance, exercising modulates glucagon‐like peptide‐1 (GLP‐1) and appetite‐regulating hormones (ghrelin and leptin). Moderate‐intensity training increases GLP‐1 response that could regulate glucose and insulin secretion [12, 13]. If exercise lasts less than 60 min, physical training of any intensity has no effect on leptin levels, but it reduces the acyl ghrelin concentration. Contrarily, if the exercise duration exceeds 60 min, it reduces leptin levels and increases the levels of total and des‐acyl ghrelin. These hormonal changes due to endurance training alter energy balance in two ways: they decrease fat accumulation and improve insulin secretion and sensitivity [14, 15]. The consideration of a broad spectrum of hormonal changes may help us to regulate energy uptake and expenditure during exercise.

At the time of physical training, drug supplementation can mediate metabolic and hormonal responses. In particular, phenolic compounds reduce oxidative stress and enhance insulin sensitivity [16]. Among these, a simple phenolic compound called protocatechuic acid (PCA) possesses notable antioxidative properties. Its antioxidant capacity can enhance antioxidant status and inhibit oxidative stress induced by exhaustive exercise [17]. In addition, PCA contributes to reducing blood glucose, HbA1c, and insulin resistance [18, 19]. It also helps in restoring hormonal profiles, including insulin, leptin, adiponectin [20], and testosterone concentrations [21]. However, there is limited evidence regarding the effects of PCA supplementation on hormonal responses during exercise. Future studies should investigate its efficacy in modulating physiological adaptations to physical training.

The beneficial effects of physical activity on body weight and glucose levels have been well studied. However, there is limited evidence on the optimal dose of physical training that is required to influence hormone levels and trigger a desired metabolic response. Therefore, it is crucial to investigate how different exercise settings affect the regulation of hormones and their subsequent impact on body weight and blood glucose levels.

## 2. Aims and Objectives

This study aimed to investigate dynamic changes in hormonal profiles, body weight, and glucose levels during physical training. Specifically, it examined the effects of moderate‐intensity exercise on hormonal status, carbohydrate metabolism, and protein metabolism in a rodent model. We hypothesized that PCA supplementation enhances glucose metabolism and counteracts the negative effect of detraining on hormonal regulation.

To achieve the aim, we defined the following objectives:

• Examine the effects of training protocols and PCA supplementation on protein and glucose metabolism.
• Assess hormonal responses to physical training and their relationship with blood glucose and body weight.
• Evaluate associations between myokine secretion, glycolysis activity, and hormonal markers of adjustment.
• Identify training parameters most strongly correlated with body weight and blood glucose levels.
• Develop and validate a machine learning regression model to predict body weight and glucose levels.

## 3. Materials and Methods

### 3.1. Study Cohort

The sample size was determined based on Mead’s resource equation [22]. We adhered to the ethical principles of the 3Rs—replacement, reduction, and refinement, in carefully selecting animals and sample size. As a result, the study cohort consisted of 28 healthy male Wistar rats (4–6 weeks old, with a body weight of 147.5 ± 28.42 g). The rats were randomly assigned to five groups according to their respective training and treatment protocols: a baseline (control) group with no exercise intervention, a group undergoing 4 weeks of exercise training (ET4), a group undergoing 8 weeks of exercise training (ET8), a group with 4 weeks of exercise training followed by 4 weeks of detraining (DT4), and a group with 4 weeks of training, 4 weeks of detraining, and additional PCA administration (PCA). This study design enabled the evaluation of exercise duration, detraining effects, and the impact of pharmacological intervention on the measured outcomes.

Prior to the experimental decapitation, all rats underwent training in their respective modes, ensuring clear differentiation between groups. Animals were euthanized by decapitation at baseline, Week 4, and Week 8. Blood and gastrocnemius muscle samples were collected 48 h after the last exercise session to minimize acute exercise effects. Blood and gastrocnemius muscle samples were collected in the morning between 9:00 a.m. and 11:00 a.m. during the light phase. Rats are nocturnal and consume most of their food during the dark phase. This is illustrated by Sidlo et al. that the male Wistar rats consumed about 80% of their total daily amount of food in the dark phase [23]. A collection of terminal blood samples and muscle samples during the light phase may reduce variability associated with both circadian and dietary intake [24]. Accordingly, all sampling in the present study was performed during the light phase in the morning to minimize the effect of diet and circadian rhythms on hormone levels.

Rats were familiarized with treadmill running during the first week by gradually increasing the speed each day. In the following 4 week training protocol, both speed and running duration were progressively increased until reaching 25 m/min for 60 min per session. After that, these settings were kept constant. The treadmill was set at a 0% slope (6 lanes; Columbus Instruments Treadmill Simplex II, Columbus, OH, USA). Details of the training protocol are described in our previous study [25].

### 3.2. Ethical Statement

The animal study protocol was approved by the Animal Research Ethics Committee of the College of Medicine and Health Sciences at United Arab Emirates University (ERA_2021_7238).

### 3.3. Animal Care and Cohort

The reporting of animal data in this study followed the ARRIVE guidelines. All animal care and experimental procedures complied with the ethical standards for animal research established by the National Institutes of Health (NIH), USA. Rats were housed in an air‐conditioned room maintained at 22 ± 2°C, with a 12 h light/dark cycle and 50% relative humidity. All animals were provided with a standard diet containing 24% protein, 8% ash, and 5% fiber.

Healthy male Wistar rats were used in this study to examine hormonal and metabolic adaptations under normal physiological conditions. While obesity and diabetes models exhibit preexisting hormonal and metabolic disturbances, including dysregulation of insulin, cortisol, ghrelin, leptin, IL‐6, and IGF‐1 [26, 27]. However, we are interested in observing the impact of different exercise settings on hormonal and metabolic responses without any pre‐existing alterations that could confound the interpretation of training and supplementation‐induced responses. Furthermore, a healthy model is better suited to observe the loss of training‐induced adaptations during the shift from physical training to detraining. A healthy model, therefore, provides the necessary physiological baseline to assess training‐induced metabolic and endocrine adaptations, as well as their loss during detraining, without interference from disease‐related dysregulation.

### 3.4. Methodology

The first objective examined the effects of physical training on skeletal muscle protein metabolism and systemic glucose regulation. We conducted treadmill‐based moderate‐intensity training (25 m/min, 60 min/day, 5 days/week) in male Wistar rats. A subset of the animals received daily oral supplementation of PCA at a dose of 100 mg/kg body weight (97% purity; Sigma‐Aldrich, MO, USA). Previous evidence has shown that PCA at a dose of 100 mg/kg body weight in rats has shown better results than 50 mg/kg in improving body weight, lipid profile, glucose levels, and insulin resistance [28]. However, a higher dose of 200 mg/kg body weight may lead to the formation of by‐products that can interfere with antioxidant activity, thereby reducing its overall effectiveness [19]. Furthermore, treatment with PCA at 100 mg/kg body weight for 4 weeks has been shown to effectively improve glucose levels and overall metabolic health [29, 30]. This dose is considered safe and has been shown to enhance endocrine function and reduce oxidative stress [21, 31].

This objective involved three sequential analyses. First, we characterized temporal changes in gastrocnemius muscle secretions using the methodology outlined in Section 3.1. Group comparisons employed one‐way analysis of variance (ANOVA) with Dunnett’s post hoc test, supplemented by Cohen’s d effect size quantification. The effect size was categorized as small (> 0.2), medium (> 0.5), large (> 0.8), or very large (> 1.3).

Second, we systematically evaluated how training within the last 1–4 weeks, detraining periods, and PCA supplementation influenced metabolic outcomes. Protein metabolism was assessed through myokine profiling (insulin‐like growth factor‐1 [IGF‐1], Interleukin‐6 [IL‐6], fibroblast growth factor 21 [FGF‐21], myostatin, irisin), while glucose metabolism was analyzed via pyruvate/glycogen levels and glycolytic enzyme activities (hexokinase [HK], phospho‐fructokinase [PFK]).

Finally, we investigated multivariate relationships between muscle‐derived factors, circulating glucose, and anthropometric measures. Correlation analyses identified statistically significant associations between these biomarkers and training parameters, providing mechanistic insights into exercise‐induced metabolic adaptations.

To address the second objective, we collected blood via incision and centrifuged it for 20 min at 15,000 × g to obtain serum. In the serum, we measured the levels of appetite‐regulating hormones (ghrelin, leptin, and glucagon‐like peptide‐1 [GLP‐1]) and steroid hormones (cortisol, testosterone, and aldosterone). The testosterone/cortisol index served as an indicator of the balance between anabolic and catabolic activity.

In this phase of the study, the computational approach was the same as in the first objective. Data were analyzed using one‐way ANOVA with Dunnett’s post hoc test for comparisons against the baseline group. Effect sizes were assessed using Cohen’s d. To evaluate hormonal responses to training, we built heatmap correlation matrices visualizing associations between hormonal profiles and training conditions. Finally, we examined correlations between hormonal profiles, body weight, and glucose levels.

The third objective focuses on the association of the hormonal profile and muscle metabolism. For the normally distributed data, we applied the Pearson correlation coefficient. If the variables did not follow the Gaussian distribution, the Spearman test was the choice. We identified strong correlations between the studied variables and created heatmaps to visualize the associations between muscle‐secreted factors and serum markers. This approach enabled us to assess the metabolic consequences of the observed changes.

The fourth objective was to identify the most effective dose for improving metabolic response. The blood glucose was measured weekly using a glucometer (Accu‐Chek Instant). Similarly, we weigh the body weight of each animal every week. To address the objective, we assessed the association of body weight and glucose tolerance with training settings and the hormonal profile. This analysis provided valuable insights for optimizing training doses to enhance metabolic health.

Achieving the fifth objective, we applied a regression model to predict body weight and glucose tolerance changes from physical training. First, machine learning algorithms were programmed to predict body weight from training duration (weeks trained/detrained), recent training history (from last day to 4 weeks), PCA dose, and dissemblance. Next, a separate model forecasted blood glucose levels with the same predictors. We evaluated three regressors—decision tree, XGBoost, and LightGBM—and measured accuracy using the mean absolute error (MAE) and MAE/ROV ratio (MAE relative to value range). Lower values of MAE indicate better predictive accuracy.

To achieve the fifth objective, we developed supervised regression pipelines to predict body weight and blood glucose from training duration, recent training load (last day and last 1–4 weeks), detraining periods, detraining ratio, and PCA dose. All predictors were numerical and missing numerical values were imputed inside the cross‐validation pipeline. Model performance was estimated with five‐fold K‐fold cross‐validation (random seed = 777) using MAE and MAE normalized to the observed range of values (MAE/ROV); lower values indicated better out‐of‐sample accuracy.

We compared eight algorithms selected to represent complementary model families: lasso regression [32], Theil–Sen regression [33, 34], support vector regression (SVR) [35], random forest [36], gradient boosting [37], XGBoost [38], LightGBM [39], and CatBoost [40]. Decision‐tree‐based models were interpreted in the context of classification and regression tree methodology [41]. Lasso assumes approximately linear additive effects and uses L1 shrinkage to reduce irrelevant predictors; Theil–Sen regression provides a robust linear estimate less influenced by outliers; SVR tests whether a maximum‐margin nonlinear surface improves prediction; random forest averages decorrelated trees to reduce variance; and boosting algorithms sequentially combine shallow trees to capture nonlinear thresholds and interactions. The models were interpreted as predictive screening tools rather than causal estimators; their significance was defined by cross‐validated predictive accuracy and convergence of important predictors across model families.

### 3.5. Serum Hormones

After decapitation, blood samples were collected immediately in sterile, dry vacutainer tubes containing a gel clot activator. After collection, blood was centrifuged at 1500 × g for 20 min, and the resulting serum was aliquoted and stored at −80°C until analysis.

Serum concentrations of cortisol, aldosterone, testosterone, ghrelin, leptin, and GLP‐1 were quantified using the enzyme‐linked immunosorbent assay (ELISA) according to the manufacturers’ protocols (see Table 1). Cortisol and aldosterone were analyzed using Thermo Scientific kits. Testosterone levels were measured with an Invitrogen kit. Ghrelin, leptin, and GLP‐1 were assayed using Elabscience Kits.

**TABLE 1:** Specifications of laboratory kits.

Kit name	Catalog no.	Producer	Sensitivity	Unit
*Myokines*				
IGF‐1	MG100	R&D Systems, Minneapolis, USA	3.5	pg/mL
Myostatin	DGDF80	R&D Systems, Minneapolis, USA	2.25	pg/mL
IL‐6	ERA32RB	Thermo Scientific, USA	15	pg/mL
FGF‐21	MBS2024083	MyBioSource, San Diego, USA	5.9	pg/mL
Irisin	MBS7205414	MyBioSource, San Diego, USA	0.1	µg/mL

*Substrates*				
Glycogen	ab65620	Abcam, Cambridge, UK		ug/uL
Pyruvate	ab65342	Abcam, Cambridge, UK		nmol/uL

*Enzymes*				
PFK	ab155898	Abcam, Cambridge, UK		mU/mL
Hexokinase	ab136957	Abcam, Cambridge UK		mU/mL

*Hormones*				
Cortisol	EIAHCOR	Thermo Scientific, USA	0.0276	ng/mL
Aldosterone	EIAALD	Thermo Scientific, USA	0.00497	ng/mL
Testosterone	ADI‐900‐065	Enzo Life Science, Farmingdale, USA	0.00567	ng/mL
Ghrelin	E‐EL‐R0842	Elabscience, Houston, USA	0.1	ng/mL
Leptin	E‐EL‐R0582	Elabscience, Houston, USA	0.1	ng/mL
GLP‐1	E‐EL‐R3007	Elabscience, Houston, USA	0.00094	ng/mL

### 3.6. Skeletal Muscle Secretions

Gastrocnemius muscles were homogenized using a rotor–stator homogenizer in cold KCl lysis buffer. The KCl lysis buffer is composed of 10 mM Tris base, pH 8, 140 mM NaCl, 300 mM KCl, 0.5% sodium deoxycholate, 0.5% SDC, 0.5% Triton X‐100, and 1 mM EDTA, supplemented with 1% protease and phosphatase inhibitor cocktail (Sigma‐Aldrich, St. Louis, MO, USA). The homogenates were centrifuged at 15,000 g for 25 min at 4°C, and the resulting supernatants were stored at −80°C.

Skeletal muscle secretions, including IGF‐1, IL‐6, FGF‐21, myostatin, and irisin, were measured with ELISA in the gastrocnemius muscle. We used commercially available kits to measure the activity of enzymes (phosphofructokinase [PFK], HK) and the levels of substrates (glycogen, pyruvate) in the same muscle. See kit specifications in Table 1.

### 3.7. Molecular Probing of Muscle Growth

Small fragments (4–6 cm^3^) of the gastrocnemius muscle were snap frozen in liquid nitrogen and cut into 5‐6 μm sections using a cryostat. The sections were later processed for immunofluorescence using antibodies against Ki‐67 according to previously published method [42]. Ki‐67 is used to identify putative proliferating satellite cells (SCs) of the gastrocnemius in response to physical exercise. Morphometric analysis was performed by counting the number of Ki‐67 immunoreactive cells per X40 microscopic field (*n* = 6).

## 4. Results

### 4.1. Role of Physical Training on Muscle and Glucose Metabolism

#### 4.1.1. Impact of Training Settings and PCA Supplementation on Muscle‐Secreted Factors

After 8 weeks of training, interleukin‐6 (IL‐6) levels increased significantly from 637.415 ± 157.01 to 1224.380 ± 376.01 pg/mL (*p* = 0.021, Cohen’s d = 2.04), indicating a large effect size (see Table 2). Longitudinal analysis revealed a progressive rise in IL‐6 concentrations across the 4‐week training period, with stronger correlations observed over time: *r* = 0.38 (*p* = 0.046) at Week 2, *r* = 0.43 (*p* = 0.022) at Week 3, and *r* = 0.47 (*p* = 0.013) at Week 4 (see Figure 1A). These results suggest a dose‐dependent relationship between training duration and IL‐6 elevation, with the association strengthening incrementally each week.

**TABLE 2:** Biochemical and muscle findings.

Estimate	CON	ET4			ET8			DT4			PCA		
		4 weeks training			8 weeks training			4 weeks training			0–4 weeks training		
								4 weeks detraining			4–8 weeks detraining and PCA		
	M_1_±SD_1_	M_2_±SD_2_	p_1−2_	d_1−2_	M_3_±SD_3_	p_1−3_	d_1−3_	M_4_±SD_4_	p_1−4_	d_1−4_	M_5_±SD_5_	p_1−5_	d_1−5_
IL‐6	637.41 ± 157.01	721.12 ± 183.12	0.984	0.49	1224.38 ± 376.01	**0.021**	2.04	919.36 ± 250.45	0.440	1.201	1091.74 ± 407.40	0.087	1.47
Myostatin	554.913 ± 93.13	385.927 ± 48.84	**0.002**	2.273	435.284 ± 48.24	**0.014**	1.613	452.342 ± 65.43	**0.041**	1.202	455.840 ± 50.56	**0.047**	0.1311
IGF‐1	157.231 ± 23.48	206.845 ± 40.33	0.131	1.504	223.311 ± 41.55	**0.012**	1.958	177.094 ± 30.87	0.729	0.669	188.930 ± 26.27	0.335	1.272
FGF‐21	58.183 ± 44.441	48.149 ± 19.391	0.990	0.293	75.676 ± 42.025	0.890	0.404	125.664 ± 55.343	0.055	1.345	88.241 ± 40.102	0.589	0.710
Irisin	0.563 ± 0.214	0.539 ± 0.082	0.998	0.148	0.561 ± 0.144	1.000	0.011	177.094 ± 0.154	0.938	0.284	0.547 ± 0.181	0.999	0.081

*Glucose metabolism markers*													
HK	8.156 ± 2.49	11.236 ± 2.13	0.531	1.330	12.340 ± 3.90	0.186	1.279	11.076 ± 3.66	0.985	0.591	12.732 ± 2.86	0.135	1.705
PFK	4.663 ± 0.22	4.727 ± 0.35	0.974	0.222	4.574 ± 0.21	0.923	0.417	4.647 ± 0.20	1.000	0.208	4.477 ± 0.13	0.404	1.033
Pyruvate	0.011 ± 0.00	0.023 ± 0.01	0.368	1.695	0.026 ± 0.01	0.203	2.118	0.034 ± 0.03	0.517	1.412	0.044 ± 0.03	**0.005**	1.314
Glycogen	0.041 ± 0.003	0.059 ± 0.010	**0.000**	2.438	0.047 ± 0.004	0.218	1.697	0.044 ± 0.004	0.740	0.849	0.048 ± 0.006	0.126	1.476

*Note:* The significant differences between the findings before and after the intervention are marked in bold.

**FIGURE 1:**
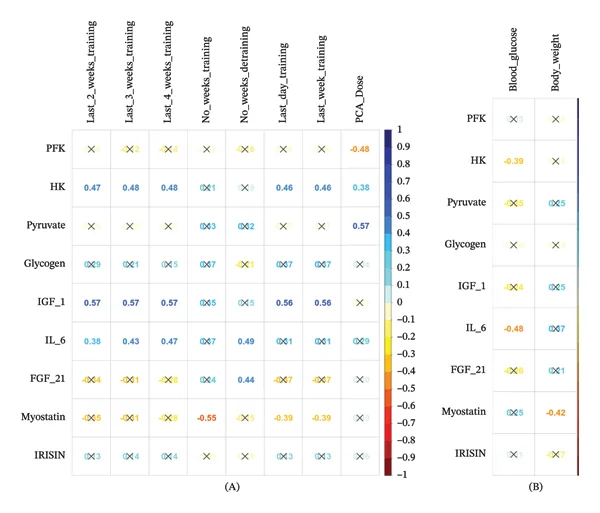
Correlation matrix of muscle secretions with training settings (A), body weight, and blood glucose (B).

Muscle insulin‐like growth factor‐1 (IGF‐1) titers exhibited a response pattern analogous to IL‐6. Following 8 weeks of training, IGF‐1 levels demonstrated a significant elevation from baseline 157.231 ± 23.48 to 223.311 ± 41.55 pg/mL; *p* = 0.012, Cohen’s d = 1.96). Analysis revealed strong positive correlations between IGF‐1 levels and training duration at both the final day and the terminal week of observation (*r* = 0.56, *p* = 0.002 for both).

The correlations suggested that even acute training interventions can modulate IGF‐1 expression. Notably, this association showed progressive intensification with sustained training over 2–4 weeks (*r* = 0.57, *p* = 0.001; see Figure 1A). The intensification indicates a time‐dependent, cumulative effect on IGF‐1 upregulation. These results suggest that IGF‐1 secretion responds to acute exercise stimuli and exhibits cumulative, time‐dependent upregulation, mirroring the pattern observed for IL‐6.

Training effectively reduces myostatin levels, showing significant decreases after 4 and 8 weeks: *p* = 0.002, *d* = 2.273 and *p* = 0.014, *d* = 1.613. Remarkably, levels stayed low even after 4 weeks of detraining: *p* = 0.041, *d* = 1.202 (see Figure 2). We found negative correlations between myostatin and recent training: *r* = −0.39, *p* = 0.041. Its association with cumulative training duration was even stronger: *r* = −0.55, *p* = 0.002. These findings demonstrate that even brief training lowers myostatin, while prolonged training yields greater reductions that promote muscle growth and combat insulin resistance.

**FIGURE 2:**
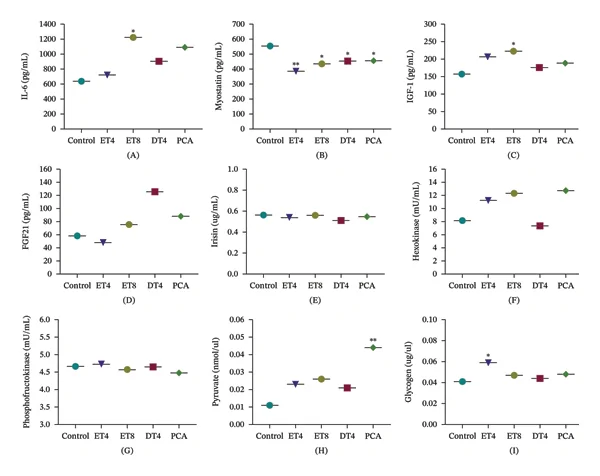
Muscle secretions and glucose metabolism markers.

Exercise also enhanced energy substrates such as glycogen. We reported a significant increase in glycogen levels from 0.041 ± 0.003 to 0.059 ± 0.010 after 4 weeks of training (*p* = 0.000, *d* = 2.438). This improvement likely reflects enhanced glucose uptake and storage in muscle tissue, driven by exercise‐induced metabolic adaptations.

PCA supplementation expressed a loose association with the myostatin titers: *p* = 0.047, *d* = 0.131. The supplementation augmented training benefits by modulating energy metabolism. Pyruvate levels increased significantly with PCA from 0.011 ± 0.004 to 0.044 ± 0.028 nmol/µL (*p* = 0.005, *d* = 1.31). The supplementation was associated positively with the HK activity, but negatively with PFK levels. The data for HK and PFK were *r* = 0.38, *p* = 0.04 and *r* = −0.48, *p* = 0.01, respectively. However, pyruvate levels have shown a positive association (*r* = 0.57, *p* = 0.002). However, the Cohen’s d value of 0.131 for the effect of PCA supplementation on myostatin concentration indicates a trivial effect size according to conventional interpretation thresholds, where *d* < 0.2 is negligible.

#### 4.1.2. Relationship Between Muscle Secretions, Glycolytic Enzymes, Blood Glucose, and Body Weight

We found that muscle secretions and glycolytic enzymes were correlated with both body weight and blood glucose levels. Notably, blood glucose showed significant negative associations with IL‐6 (*r* = −0.48, *p* = 0.01) and HK (HK; *r* = −0.39, *p* = 0.039), suggesting that higher levels of these factors are linked to lower blood glucose. These findings imply that training‐induced elevations in IL‐6 and HK activity may facilitate glucose utilization, thereby promoting reductions in blood glucose levels through enhanced metabolic activity.

Additionally, myostatin displayed a negative correlation with body weight (*r* = −0.42, *p* = 0.027) (Figure 1B). Given that myostatin functions as an inhibitor of skeletal muscle growth, its suppression during training is likely a key factor in promoting muscle hypertrophy. This reduction in myostatin in response to exercise may contribute to increased muscle mass and improved regulation of body weight, further emphasizing the physiological benefits of exercise‐induced molecular changes.

### 4.2. Changes in Hormonal Status in Response to Training Settings, Body Weight, and Glucose Levels

#### 4.2.1. Hormonal Adaptation to Training

Our results revealed that 8 weeks of exercise training (ET8) significantly reduced cortisol levels from 1.380 ± 0.660 to 0.517 ± 0.307 ng/mL, *p* = 0.028, *d* = 1.677 (see Table 3). Interestingly, the testosterone‐to‐cortisol (T/C) ratio increased significantly with 8 weeks of training. The T/C ratio was higher at 8 weeks (1.311 ± 0.617, *p* = 0.009, *d* = 2.465) compared to the control 90.211 ± 0.133). In addition, the level of leptin correlated with the duration of exercise training. It elevated from 0.323 ± 0.065 to 0.708 ± 0.270 ng/mL (*p* = 0.014, *d* = 0.917) and 1.578 ± 0.220 ng/mL (*p* = 0.001, *d* = 3.098) by the end of the 4th and 8th weeks, respectively. When the exercises were stopped, the levels of leptin reduced to 0.674 ± 0.164 ng/mL, but remained greater than at the baseline (*p* = 0.010, *d* = 0.896). However, the administration of PCA supplementation during the detraining period significantly boosted leptin levels again to 1.199 ± 0.103 (*p* = 0.001, *d* = 2.299). Higher levels of leptin may stimulate appetite and maintain energy balance after exercising.

**TABLE 3:** Hormonal findings.

Estimate	CON	ET4			ET8			DT4			PCA		
		4 weeks training			8 weeks training			4 weeks training			0–4 weeks training		
								4 weeks detraining			4–8 weeks detraining and PCA		
	M_1_±SD_1_	M_2_±SD_2_	p_1−2_	d_1−2_	M_3_±SD_3_	p_1−3_	d_1−3_	M_4_±SD_4_	p_1−4_	d_1−4_	M_5_±SD_5_	p_1−5_	d_1−5_
Cortisol	1.380 ± 0.660	0.797 ± 0.244	0.2569	1.476	0.517 ± 0.307	**0.0227**	1.677	1.096 ± 0.676	0.7008	0.425	0.689 ± 0.381	0.0803	1.282
Aldosterone	0.221 ± 0.162	0.287 ± 0.083	0.950	0.513	0.359 ± 0.302	0.522	0.569	0.207 ± 0.080	1.000	0.110	0.183 ± 0.144	0.988	0.248
Testosterone	0.272 ± 0.183	0.440 ± 0.267	0.771	0.734	0.574 ± 0.338	0.229	1.111	0.498 ± 0.099	0.453	1.536	0.531 ± 0.362	0.343	0.903
T/C	0.211 ± 0.133	0.537 ± 0.214	0.771	1.830	1.311 ± 0.617	**0.009**	2.465	0.627 ± 0.429	0.497	1.310	0.999 ± 0.735	0.073	1.492
T/A	1.558 ± 0.817	1.559 ± 0.818	1.000	0.001	2.975 ± 2.305	0.552	0.819	2.897 ± 1.752	0.596	0.980	3.894 ± 2.468	0.172	1.271
A/C	0.150 ± 0.091	0.375 ± 0.098	0.916	2.379	0.957 0.984	0.061	1.155	0.321 ± 0.290	0.944	0.796	0.275 ± 0.217	0.981	0.751

*Nonsteroid hormones*													
Grelin	0.591 ± 0.082	0.527 ± 0.123	0.727	0.612	0.465 ± 0.125	0.129	1.192	0.536 ± 0.059	0.731	0.770	0.548 ± 0.095	0.856	0.485
Leptin	0.323 ± 0.065	0.708 ± 0.270	**0.014**	0.917	1.578 ± 0.220	**0.001**	3.098	0.674 ± 0.164	**0.010**	0.896	1.199 ± 0.103	**0.001**	2.299
GLP‐1	0.026 ± 0.009	0.025 ± 0.013	0.999	0.089	0.027 ± 0.010	0.999	0.105	0.020 ± 0.006	0.565	0.784	0.025 ± 0.006	0.999	0.131

*Note:* The significant differences between the findings before and after the intervention are marked in bold.

The levels of aldosterone also correlated with the duration of training. The association was remarkable at the end of the second week (*r* = 0.37, *p* = 0.049), and it strengthened further by the end of the third and fourth weeks (*r* = 0.38, *p* = 0.047). This rise in aldosterone levels during training may help the body retain water, maintain blood pressure, and balance sodium and potassium levels.

We found a negative correlation between the level of cortisol and the amount of training within the last day and last week of the experiment (*r* = −0.40, *p* = 0.036; *r* = −0.40, *p* = 0.036). The negative association of cortisol secretion with exercise duration strengthened progressively with r‐coefficients reaching −0.42, −0.43, and −0.43 at the end of the second, third, and fourth weeks (*p* = 0.027, 0.024, and 0.023, respectively). We also noted a weak negative association between the total number of training weeks and cortisol: *r* = −0.38, *p* = 0.048.

Ghrelin levels followed a similar trend. The level of ghrelin correlated negatively with the amount of training within the last day and last week of the experiment (*r* = −0.38, *p* = 0.048; *r* = −0.38, *p* = 0.048). The negative association persisted over time: *r* = −0.40, *p* = 0.034; *r* = −0.42, *p* = 0.027; and *r* = −0.42, *p* = 0.025 for the second, third, and fourth weeks, correspondingly. These findings indicate that as training duration increased, the negative correlation between cortisol and ghrelin became more pronounced. This suggests an adaptive response to sustained physical activity. Physical training may potentiate hormonal secretions that help maintain positive energy balance and regulate body weight. After a course of exercise training, the levels of cortisol and ghrelin may reduce due to the adjustment.

PCA supplementation showed a positive association with the T/C ratio: *r* = 0.40, *p* = 0.035 (see Figure 3). Hence, PCA may enhance testosterone levels while reducing cortisol levels. The drug supplementation could potentially reduce physiological stress. It might also improve energy metabolism at the time of restoration, thus contributing to a positive hormonal response to sports activity.

**FIGURE 3:**
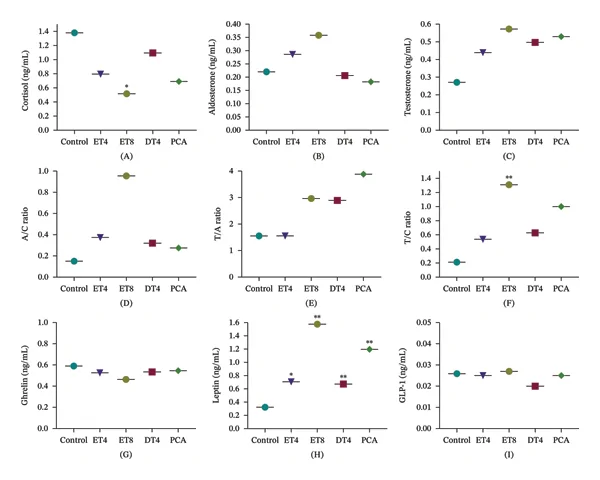
Impact of training on hormonal levels.

#### 4.2.2. Hormonal Regulation of Glucose and Body Weight

Our study revealed no association between serum hormone levels, blood glucose, and body weight (Figure 4B). The absence of correlation may result from differences in the timing of responses between hormonal changes and metabolic markers. Endocrine adaptations often occur more rapidly than the physiological changes required to influence body weight and blood glucose levels. As a result, this temporal disconnect may obscure transient physiological and metabolic responses to exercise, particularly in short‐term studies. Therefore, it is important to consider both timing and regulatory complexity when interpreting hormone–metabolism interactions in animal models.

**FIGURE 4:**
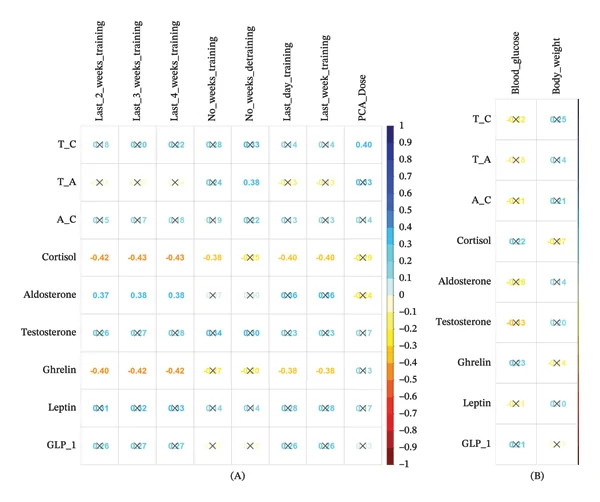
Correlation matrix of serum hormones with training settings (A) and body weight and blood glucose (B).

### 4.3. Association of Hormonal Markers With Muscle Secretions

We observed a correlation between hormonal status markers and muscle‐secreted factors. For instance, IL‐6 was negatively associated with ghrelin: *r* = −0.52, *p* = 0.005. Ghrelin as a hormone and IL‐6 as a muscle secretion regulate appetite, energy expenditure, and body composition [43, 44]. The inverse relationship between IL‐6 and ghrelin may contribute to appetite reduction.

IGF‐1 correlated positively with aldosterone (*r* = 0.48, *p* = 0.01) and a negatively with cortisol (*r* = −0.50, *p* = 0.006). It also showed a tight association with the aldosterone‐to‐cortisol (A/C) ratio: *r* = 0.48, *p* = 0.012 (see Figure 5). The positive correlation between IGF‐1 and aldosterone suggests a potential role of IGF‐1 in enhancing aldosterone‐mediated sodium balance and metabolic regulation. In contrast, its negative correlation with cortisol implies that lower cortisol levels may promote IGF‐1 production. A reduction in cortisol levels mitigates stress, improves insulin sensitivity, and reduces metabolic disturbances.

**FIGURE 5:**
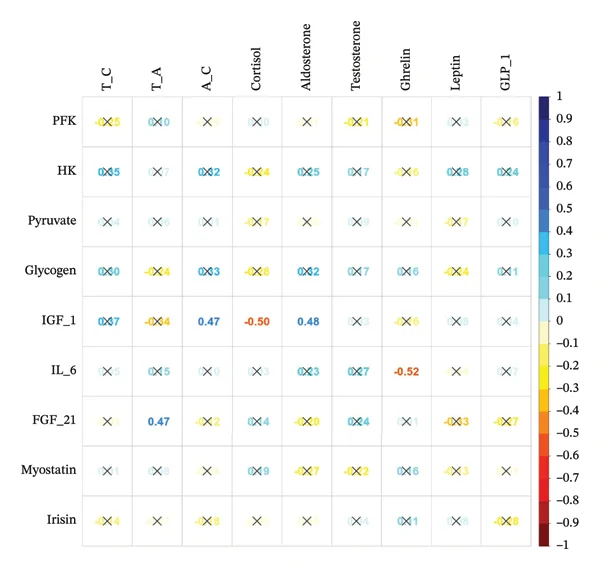
Correlation matrix of hormone status with muscle secretions and glycolytic enzymes.

The secretion of FGF‐21 was in concordance with the T/A ratio: *r* = 0.47, *p* = 0.012. FGF‐21 may enhance metabolic adaptation to exercise training by modulating the T/A ratio, thereby promoting favorable shifts in energy utilization. FGF‐21 plays a key role in improving glucose homeostasis, lipid metabolism, and insulin sensitivity. The correlation observed in our study suggests that FGF‐21 may act as a compensatory mediator, facilitating the metabolic benefits associated with sustained physical activity.

### 4.4. Modulation of Body Weight and Glucose Levels With Different Training Settings

The blood glucose level did not correlate with the duration of training within the last day of the experiment (see Figure 6). It also showed a miscorrelation with the amount of physical activity performed within 1–4 weeks before the incision. However, the total number of training weeks expressed a modest negative association with the glucose concentration (*r* = −0.56, *p* < 0.002). Contrarily, the number of weeks spent without physical activity showed a negative linear relationship with the blood glucose (*r* = −0.51, *p* < 0.005).

**FIGURE 6:**
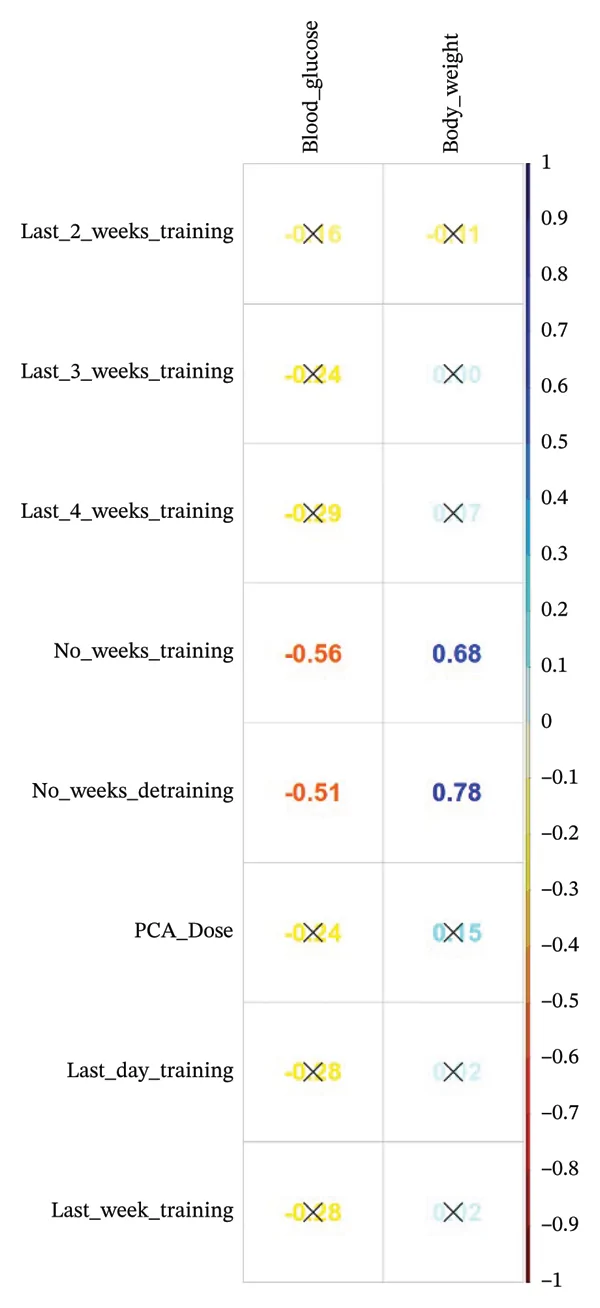
Relationship between different levels of training with blood glucose and body weight.

The study reveals a beneficial effect of exercise training: practicing physical activities lowers blood glucose levels. The link between the glucose levels and detraining time has no clear reason. Limitations of the study design may account for the noninterpretable findings. Nevertheless, the prolonged effect of exercise may be related to improvements in insulin sensitivity and glucose uptake that persist beyond the training period.

Similarly, physical training for 1–4 weeks showed no association with body weight. However, the total number of training weeks was positively correlated with body weight: *r* = 0.68, *p* < 0.002 (see Figure 6). However, the number of weeks without training also demonstrated a positive association with body weight: *r* = 0.78, *p* < 0.005. These findings suggest that exercise training leads to an increase in body weight, likely due to gains in muscle mass. Notably, the body weight remained elevated after the exercises were stopped. The supposed reasons are sustainable muscle mass gains due to aerobic exercise.

### 4.5. Prediction of Body Weight and Glucose Tolerance in Response to Different Training Settings

#### 4.5.1. Estimating Body Weight Changes in Response to Training

We evaluated multiple machine learning models (ML) to predict body weight. The XGBoost regressor demonstrated the best performance, achieving the MAE/ROV ratio of 9.31%. Gradient boosting and random forest showed almost similar accuracy: MAE/ROV = 9.42% and 9.58%, respectively. Other models (CatBoost, LGBM regressor) also performed competitively (see Table 4). In contrast, SVR was unreliable: MAE/ROV = 17.97%. Overall, the ML regressors yielded accurate predictions for the body weight due to the reliable predictors at the input to the models.

**TABLE 4:** Performance of regression models reflecting change in body weight and serum glucose concentration.

Regressors	Body weight model		Glucose level model	
	MAE	MAE/ROV (%)	MAE	MAE/ROV (%)
CatBoost	26.54	10.02	6.36	9.64
Random forest	25.4	9.58	6.59	9.98
XGBoost	24.68	9.31	6.83	10.35
LGBM	26.23	9.9	6.37	9.65
Gradient boosting	24.95	9.42	6.98	10.58
Theil–Sen	26.25	9.91	10.06	15.24
Support vector	47.61	17.97	7.1	10.76

Abbreviations: MAE, mean absolute error; ROV, range of values.

For the best‐performing model (XGB regressor), we further explored the impact of various training settings on the body weight. To address the task, we employed tree‐based feature importance analysis (see Figure 7A). The most important predictor of body weight was the number of weeks of exercise training (No_weeks_training). The second most important feature was the number of weeks of detraining (No_weeks_detraining). Our results demonstrated that the cumulative duration of both training and subsequent detraining periods is the critical determinant of body weight. This underscores the lasting physiological impact of physical activity on body composition.

**FIGURE 7:**
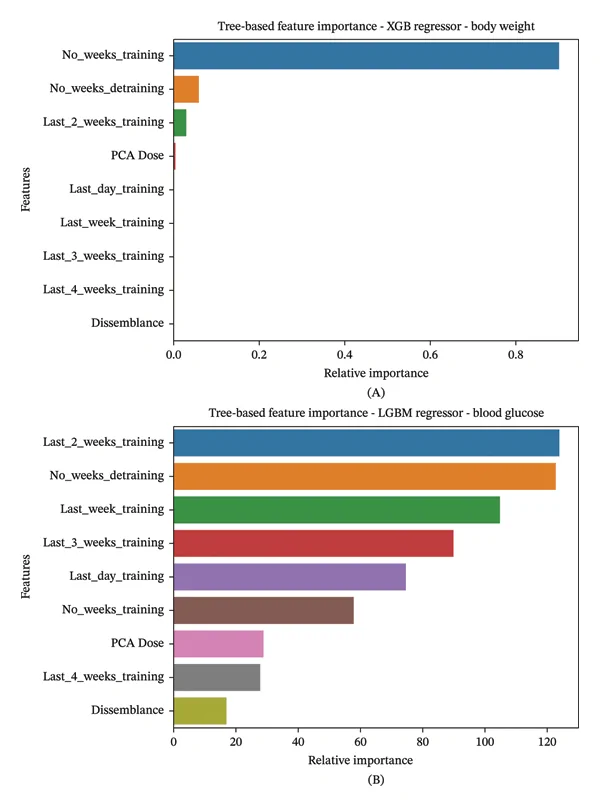
Feature importance in the prediction of body weight (A) and blood glucose (B).

#### 4.5.2. Prognosticating Blood Glucose Level From Training Settings

Among the models trained to predict blood glucose, CatBoost and LightGBM achieved the best performance, with the MAE/ROV of 9.64% and 9.98%, respectively (see Table 4). Other regressors (random forest, XGBoost, and gradient boosting) exhibited slightly higher error rates, while traditional linear models did not perform well: MAE/ROV = 15.24% for Theil–Sen regression. Overall, tree‐based methods, particularly CatBoost and LightGBM, provided better accuracy in predicting blood glucose.

The LightGBM regressor model identified distinct patterns in feature importance for predicting blood glucose levels, with recent training history emerging as the most influential predictor. We noted that the last 2 weeks of training were the most important predictors, and the last week of training ranked among the top three features for predicting maximum blood glucose in male Wistar rats (see Figure 7B). The findings suggest that the settings of the recent training (last 2 weeks) have a strong impact on blood glucose regulation.

### 4.6. Effect of Exercise Training on Skeletal Muscle Satellite Cells

It is well known that SCs of skeletal muscle are activated following physical exercise. Ki‐67, a protein found only in actively dividing cells, was used as a marker of activated SCs in the gastrocnemius. The number of Ki‐67‐positive SCs was significantly (0.0001) higher in the gastrocnemius of rats subjected to 4–8 weeks of moderate intensity physical training, compared to control groups (Figures 8 and 9).

**FIGURE 8:**
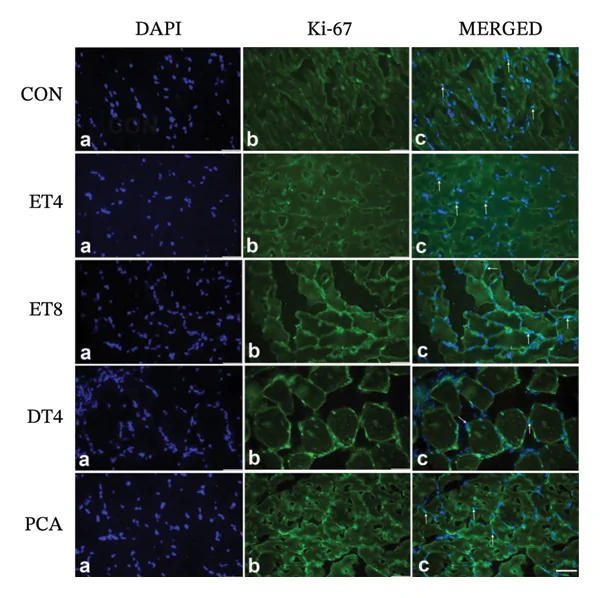
Images of sections of the gastrocnemius muscle processed for immunohistochemistry using antibodies against Ki‐67 (a marker for proliferative cells such as satellite cells of skeletal muscle). In addition, DAPI (4,6‐diamidino‐2‐phenylindole), a blue colored dye that stains DNA of nuclei, was also used. a: DAPI‐stained nuclei of skeletal muscle cells of CON (control), ET4 (4 weeks training), ET8 (8 weeks training), DT4 (4 weeks training followed by 4 weeks detraining) and PCA (4 weeks training + 4–8 weeks detraining and PCA) rats. b: Ki‐67 positive satellite cells of skeletal muscles of CON, ET4, ET8, DT4, and PCA in rats. c: merged images (cyan color) of a and b for CON, ET4, ET8, DT4, and PCA groups showing satellite cells (white arrows). *n* = 6 and scale bar = 25 μm.

**FIGURE 9:**
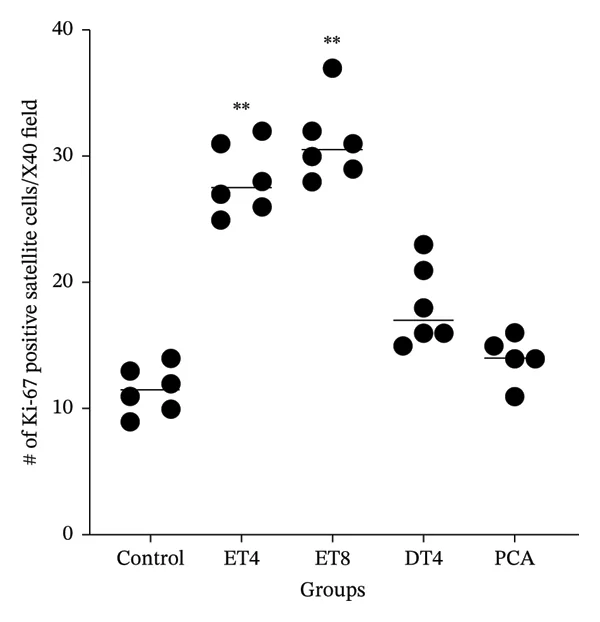
Morphometric analysis of Ki‐67 immunoreactive cells in the gastrocnemius muscle of rats in CON, ET4, ET8, DT4, and PCA groups. The number of Ki‐67 immunoreactive cells was significantly (0.0001) higher in ET4 and ET8 compared to controls. *n* = 6. ^∗∗^ET4 and ET8 versus control.

## 5. Discussion

### 5.1. Metabolic Outcomes of Training

Physical training modulates muscle secretions, particularly IL‐6, IGF‐1, and myostatin, which play critical roles in regulating glucose metabolism and body weight. In our study, regular exercise training significantly increased IL‐6 and IGF‐1 levels while concurrently reducing myostatin concentrations, thereby promoting muscle mass, lowering blood glucose levels, and improving overall metabolic health. Notably, these beneficial effects were evident even after short‐term training and became more pronounced with longer training durations. In addition, PCA supplementation modulates HK, PFK, and pyruvate, thereby improving metabolism and overall metabolic health.

The metabolic improvements induced by exercise are physiologically significant, as physical inactivity may lead to dysregulated glucose metabolism and could result in the progression of metabolic disease. This dysregulation is strongly associated with hormonal imbalances and increased disease risk [45]. Importantly, regular exercise training effectively counteracts these effects by improving body weight management, lowering blood glucose, and increasing insulin sensitivity, thus reducing the risk of type 2 diabetes [46]. These benefits can be maximized by adhering to the World Health Organization (WHO) recommendation of at least 150–300 min of moderate‐intensity aerobic activity per week [47].

Our findings provide mechanistic evidence and reinforce the importance of regular physical activity, demonstrating that exercise can beneficially influence body composition, muscle metabolism, and hormonal regulation. However, the extent of these benefits appears to be dependent on both the duration and intensity of training. Therefore, we observed that different training durations produced distinct effects on hormonal profiles and muscle secretions, ultimately shaping metabolic outcomes.

#### 5.1.1. Training‐Induced Muscle Secretions and Metabolism

In our study, the IL‐6 concentration exhibited a positive association with the number of training sessions. Although IL‐6 is classified as a proinflammatory cytokine, it also exhibits anti‐inflammatory properties and may help mitigate inflammation during the acute phase response [48, 49]. In response to exercise, IL‐6 levels can increase without muscle damage, rising up to 100‐fold before rapidly declining post‐exercise [50]. However, following ultra‐endurance exercise, IL‐6 levels remain elevated for up to 48 h [51]. The degree of IL‐6 elevation is influenced by several factors, including exercise intensity, duration, and an individual’s exercise capacity [52]. Short‐term moderate‐intensity training leads to an increase in IL‐6 levels [53], while long‐term (> 12 weeks) could result in a reduction, which might be due to body adaptation to exercise [54].

We observed a significant increase in skeletal muscle IL‐6 levels following training. Consistent with this finding, a previous study reported an increase in IL‐6 following 8 weeks of endurance training [55]. This elevation may be beneficial, as IL‐6 plays a key role in regulating glucose metabolism, preserving glycogen content, and enhancing insulin sensitivity via AMP‐activated protein kinase (AMPK) activation [56]. Furthermore, we also observed that the increase in IL‐6 levels was associated with improved blood glucose regulation. This aligns with in vitro evidence showing that IL‐6 enhances glucose uptake by activating the AMPK and phosphatidylinositol 3‐kinase (PI3K) pathways [57].

The current study demonstrates that muscle IGF‐1 levels increase significantly following 8 weeks of training. The IGF‐1 concentrations show strong temporal correlations that intensify with training duration. IGF‐1 exerts anabolic effects, and its upregulation in response to exercise enhances insulin sensitivity, maintains glucose homeostasis, and improves muscle mass and function [58, 59]. It promotes muscle hypertrophy by activating PI3K/protein kinase B (Akt) signaling pathways [58]. A single bout of moderate‐intensity aerobic training [59] and prolonged training [60] both elevate IGF‐1 levels. IGF‐1 levels remain elevated for up to 24 h after exercise [61]. In the present study, we observed a significant increase in IGF‐1 levels following a single bout of moderate‐intensity exercise, with further improvement after 4 weeks of training. This aligns with previous findings by Pranoto et al., who demonstrated that a single session of moderate‐intensity exercise elevates IGF‐1 levels [59]. Moreover, aerobic exercise training for 4 weeks has been shown to significantly upregulate IGF‐1, contributing to improvements in skeletal muscle mass, function, and metabolic responses [62, 63].

On the other hand, myostatin is a negative regulator of muscle growth, differentiation, and insulin‐signaling pathways. We observed a negative relationship between myostatin levels and body weight, as suppressing myostatin secretion can increase skeletal muscle mass and strength. Physical inactivity and a sedentary lifestyle are associated with increased myostatin protein concentrations [64]. Therefore, increasing physical activity plays a key role in reducing myostatin levels. Interestingly, our results revealed a significant negative association between myostatin and when training duration is less than 2 weeks, as well as the total number of training weeks. These findings suggest that even short‐term training effectively reduces myostatin levels. However, a greater reduction was observed with the cumulative effect of prolonged training. In line with this, moderate‐intensity exercise has been shown to reduce myostatin levels and enhance IGF‐1 levels [65], contributing to improved glucose metabolism, muscle mass, and body weight. In our study, exercise training significantly reduced myostatin levels, with effects lasting even after detraining.

#### 5.1.2. Role of Training and Protocatechuic Acid in Energy Metabolism

We showed that training boosts IL‐6 and HK activity, enhancing glucose utilization and lowering blood glucose. Myostatin suppression during exercise counteracts its muscle growth inhibition. This myostatin reduction promotes skeletal muscle mass accumulation. Together, these mechanisms improve both glucose metabolism and body weight regulation.

Beyond the effect of exercise, we also found that PCA supplementation modulates key metabolic enzymes such as HK and PFK. HK is an enzyme that catalyzes the first step of glycolysis by phosphorylating glucose. Our results reported that an increase in HK improved blood glucose levels. These findings align with a previous study showing that exercise enhances HK, which in turn boosts glucose uptake by upregulating glucose transporter type 4 (GLUT‐4) [66]. On the other hand, PFK is considered a rate‐limiting enzyme in the glycolysis pathway. While exercise alone significantly influenced HK activity, we found that PCA supplementation, although achieving statistical significance, had a negligible impact compared to exercise. However, PCA supplementation demonstrated a greater effect on the activity of HK and PFK and pyruvate levels, suggesting a metabolic adaptation involving a reduced reliance on glycolysis and a shift toward alternative energy substrates [67].

Moreover, we found a positive association between PCA supplementation and HK activity while showing a negative association with PFK. This metabolic shift suggests that PCA may promote greater utilization of fatty acids and other alternative energy sources, thereby sparing glucose and enhancing overall energy efficiency. Supporting this, previous research has shown that PCA supplementation enhances antioxidant capacity and activates AMPK pathways [68]. Notably, AMPK activation is known to regulate fatty acid metabolism, stimulate mitochondrial biogenesis, and improve aerobic capacity [69].

### 5.2. Impact of Training Settings on Hormonal Status

We observed an elevation in aldosterone levels with 8 weeks of physical training. Aldosterone is a steroid hormone produced by the adrenal cortex, playing a crucial role in maintaining electrolyte balance and fluid homeostasis. Elevated aldosterone levels help to regulate sodium retention, thereby regulating blood pressure and blood flow to ensure adequate oxygen delivery to muscles during exercise [70]. Significant increases in aldosterone levels have been reported in response to aerobic exercise [71, 72], which aligns with our findings. This increase might maintain electrolyte and fluid balance during exercise. Contrarily, a study involving 12 weeks of voluntary exercise in hypertensive male Sprague Dawley rats reported a significant reduction in plasma aldosterone levels [73]. This is not aligned with our findings, but this discrepancy may be attributed to differences in training durations, type of training (voluntary vs. structured), and animals’ physiological status (hypertensive vs. healthy), which could influence aldosterone regulation. The reduction in aldosterone may also reflect improved fluid balance and cardiovascular adaptations associated with long‐term training.

Furthermore, the present study found that serum cortisol levels decreased significantly following 8 weeks of training. Cortisol is a stress hormone that plays a crucial role in regulating metabolism, stress, and inflammatory response. Increased cortisol levels are associated with higher body mass index, body weight, fat mass, and waist circumference. Additionally, prolonged elevation of cortisol levels is associated with insulin resistance and visceral obesity, which can contribute to metabolic syndrome [74]. Exercise is an effective intervention for managing overweight, obesity, and stress by reducing cortisol levels [75]. We found a negative relationship between training settings and cortisol levels. This is consistent with previous evidence showing that moderate‐intensity aerobic training reduces cortisol levels, thereby helping to manage overweight and stress response [76]. Similarly, it was reported that training‐induced reduction in cortisol levels improves energy metabolism, markers of overweight, and stress response [76].

In our study, 4 and 8 weeks of training elevated testosterone levels. Testosterone is a crucial hormone in carbohydrate, fat, and protein metabolism and plays an important role in regulating body fat composition and muscle mass in males [77]. Low testosterone levels are associated with increased fat mass, reduced insulin sensitivity, impaired glucose tolerance, and dyslipidemia, leading to metabolic disease [78]. Our findings demonstrate an increase in testosterone levels with exercise training, but it did not reach statistical significance. However, we reported a significant positive association between PCA supplementation and the testosterone‐to‐cortisol ratio (T/C), showing a potential role of PCA in improving testosterone levels while reducing cortisol. Evidence indicates that PCA lowers blood glucose levels, prevents body weight gain, reduces oxidative stress and inflammation, and restores testosterone levels in diabetic rats [21]. Additionally, another study reported that PCA enhances antioxidant defense, decreases oxidative stress, and increases testosterone levels in Wistar rats [79]. These findings, consistent with our results, suggest that PCA supplementation during detraining could regulate hormonal balance, improve metabolism, and manage body weight.

We observed an elevation in leptin levels and a reduction in ghrelin levels following 8 weeks of training. Leptin, a satiety hormone secreted by adipose tissue, plays a key role in regulating energy balance and metabolic processes. It acts on the hypothalamus in the brain, which is responsible for body weight regulation. When fat stores increase, leptin levels rise, which suppresses appetite and increases energy expenditure, helping to reduce body weight. Conversely, when fat mass decreases, leptin levels fall, which stimulates appetite and decreases energy expenditure. This helps the body to restore lost fat. In this way, leptin maintains adipose tissue mass and regulates body weight [80]. In our study, we reported a significant increase in leptin levels after training, which may suppress appetite and increase energy expenditure. This is aligned with previous findings demonstrating that regular aerobic exercise training on a treadmill for 30 min, five times a week for 6 weeks, elevated blood leptin levels in female Wistar rats [81]. Contrarily, other findings have shown that 12 weeks of aerobic training reduced leptin levels, whereas a subsequent 4 weeks of detraining led to an increase in leptin concentrations [82]. On the other hand, PCA supplementation during detraining increases the leptin levels, showing that the phenolic compound also helps in regulating appetite and increasing energy expenditure. A previous finding by Hussein et al. reported an elevation in blood leptin in mice after administration of mate tea rich in polyphenols [83]. Similarly, Brazilian green propolis was found to dramatically boost leptin expression when administered in vitro to the 3T3‐L1 cell line and in vivo to C57BL/6 mice [84]. A systematic analysis of green tea extract rich in polyphenols increases leptin levels if consumed for more than 12 weeks [85]. This shows the importance of exercise training and PCA supplementation on the leptin levels.

On the other hand, ghrelin, often referred to as the ‘hunger hormone,’ is produced by endocrine cells in the gastrointestinal tract. It stimulates appetite, increases food intake, and plays a key role in maintaining energy balance and body weight. A decrease in ghrelin hormone suppresses appetite and supports weight management [44]. Dysregulation of ghrelin has been linked to overweight and obesity [86, 87]. Our findings revealed a negative correlation between ghrelin levels and training duration. This association strengthens as training weeks increase. Consistent with our results, a previous study reported a reduction in delta acylated ghrelin concentrations following both moderate‐ and vigorous‐intensity exercise. Additionally, vigorous running for 45 or 90 min significantly reduced delta acylated ghrelin concentrations (45 min: ES = 0.77, *p* < 0.001; 90 min: ES = 0.68, *p* < 0.001) [88]. Extending exercise duration may prolong the transient suppression of ghrelin and hunger.

### 5.3. Endocrine and Muscular Crosstalk: Interaction of Hormonal Profile and Muscle Secretions

Physical exercise modulates various hormones and myokines that are critical for maintaining metabolic health. Ghrelin, a hormone, and interleukin‐6 (IL‐6), a myokine, both play important roles in regulating body weight and energy balance [44, 86]. Our study found that physical training increased IL‐6 concentrations, which in turn led to a reduction in ghrelin levels. This aligns with findings from Hunschede et al., who reported that exercise suppresses appetite by increasing IL‐6 and lowering ghrelin levels [89]. This emphasizes the potential role of training in regulating muscle‐secreted factors and appetite hormones, potentially contributing to appetite regulation and body weight management.

Training‐induced IGF‐1 has been positively associated with aldosterone and negatively associated with cortisol. Previous studies have shown that aldosterone enhances IGF‐1 signaling, insulin receptor substrate, and Akt activation [90]. Activation of these pathways results in improved glucose uptake and insulin sensitivity. Furthermore, IGF‐1 helps in regulating sodium balance by enhancing both basal and aldosterone‐stimulated sodium (Na+) transport in the aldosterone‐sensitive distal nephron [90]. It may also facilitate aldosterone’s effect on Na+ transport by maintaining a basal level of active PI3K [90]. In contrast, high cortisol concentrations reduce skeletal IGF‐1 production by decreasing IGF‐1 transcript levels [91]. Similarly, we observed an inverse relationship between training‐induced IGF‐1 levels with cortisol. These findings suggest that training‐induced modulation of IGF‐1 may enhance metabolic and electrolyte balance by promoting aldosterone and reducing cortisol.

Furthermore, our findings demonstrate a positive correlation between FGF‐21 and the T/A ratio, highlighting the potential role of FGF‐21 in regulating the balance between testosterone and aldosterone, given that testosterone is known to improve insulin sensitivity and energy metabolism [77]. While aldosterone, although beneficial for sodium homeostasis and IGF‐1 signaling, has been shown to impair insulin sensitivity in skeletal muscle and adipose tissue under certain conditions [92], this association indicates the important role of FGF‐21 in promoting hormonal balance to enhance metabolic response. These results align with previous studies suggesting that FGF‐21 improves glucose and lipid metabolism, reduces body weight and fat mass, and alleviates insulin resistance [93, 94]. Moreover, the potential of FGF‐21 in modulating the T/A could serve as a key mechanism for improved metabolic health. Future research is warranted to explore the mechanistic pathways underlying the crosstalk between FGF‐21, testosterone, and aldosterone.

### 5.4. Exercise‐Induced Changes in Body Weight and Glucose Levels

We found a positive association between the total weeks of training and lower glucose levels. This indicates that cumulative exercise training potentially decreases blood glucose levels. Regular exercise training enhances skeletal muscle insulin sensitivity, increases the expression of GLUT‐4, and promotes glucose uptake, which in turn reduces blood glucose levels [95]. Additionally, adaptations in muscle substrate metabolism and oxidative capacity in response to exercise contribute to improved overall metabolic health and better glycemic control [96]. A meta‐analysis showed that aerobic exercise significantly affects glycemic levels in prediabetic subjects. Moreover, aerobic exercise training reduced body mass index, fasting blood glucose, 2 h plasma glucose, and glycated hemoglobin (HbA1c). The extent of these improvements is likely to depend upon the duration of aerobic exercise intervention [97]. Similarly, we found that a greater cumulative duration of training was associated with a greater reduction in blood glucose levels.

Additionally, another previous study has shown that aerobic and strength training (9 months) causes a moderate reduction in body mass index and fasting plasma glucose and a greater reduction in postprandial glucose. While detraining (3 months) reversed the beneficial effect of training on postprandial glucose in women with type 2 diabetes [98], however, we have observed that the blood glucose levels remained low even after 4 weeks of detraining. This persistence of improved glucose levels aligns with findings that elevated glucose uptake can last for several hours following exercise [99], suggesting that the body may require more time to fully lose the metabolic benefits of training.

In addition to metabolic improvements, exercise training also induces important structural adaptations in skeletal muscle. Skeletal muscle is a dynamic tissue that can undergo hypertrophy or atrophy in response to physiological factors such as growth and aging, as well as lifestyle factors including physical activity and nutrition [100]. Aerobic exercise training could enhance muscle mass, mitochondrial biogenesis, and muscle protein synthesis, which could result in hypertrophy of myofiber and whole muscle [101]. In our study, we found that total weeks of exercise training significantly increase body weight, which might be because of an increase in muscle mass. This is aligned with the previous finding of Jang et al. who demonstrated that 12 weeks of training significantly (*p* < 0.01) increased body weight in male rats. While body weight remained similar in the control group after 6 weeks and 12 weeks of detraining [102]. The metabolic and structural adaptations in skeletal muscle induced by training may persist even during detraining, contributing to sustained changes in body composition.

### 5.5. Moderating Body Weight and Glucose Tolerance With Exercises

The models in this study were evaluated to clarify whether body weight and blood glucose could be predicted consistently from training exposure, detraining, and PCA dose. For body weight, the lowest numerical error was observed for lasso regression (MAE = 24.44 g; MAE/ROV = 9.22%), while XGBoost was the best‐performing tree‐ensemble model (MAE = 24.67 g; MAE/ROV = 9.31%). Gradient boosting (25.12 g; 9.48%), random forest (25.58 g; 9.65%), LightGBM (26.16 g; 9.87%), and CatBoost (26.61 g; 10.04%) produced similar performance, whereas Theil–Sen (30.02 g; 11.33%) and SVR (47.64 g; 17.98%) were less suitable for this outcome. XGBoost was therefore used for feature‐importance interpretation because it combined near‐best accuracy with nonlinear interaction modeling; total weeks of training emerged as the dominant predictor, followed by detraining indicators.

For blood glucose, LightGBM achieved the lowest error (MAE = 6.36 mg/dL; MAE/ROV = 9.64%), followed closely by CatBoost (6.44 mg/dL; 9.76%), random forest (6.54 mg/dL; 9.91%), lasso regression (6.57 mg/dL; 9.95%), XGBoost (6.81 mg/dL; 10.32%), gradient boosting (6.98 mg/dL; 10.58%), and SVR (7.11 mg/dL; 10.77%). Theil–Sen regression showed lower suitability for glucose prediction (13.33 mg/dL; 20.20%). These results indicate that recent training load and cumulative training exposure have predictive value across model families rather than only within a single algorithm.

The significance of the machine‐learning analysis was therefore predictive and biological. Predictively, the convergence of tree‐based, regularized linear, and kernel‐based models supports the robustness of the main signal. Biologically, the models separated longer‐term body‐weight adaptation, best captured by cumulative training weeks, from the shorter‐term glucose response, best captured by recent training history. These findings agree with prior studies applying XGBoost to weight‐status prediction [103] and LightGBM to glucose or diabetes prediction [104, 105], while extending those approaches to an experimental training/detraining setting in which short‐term exercise exposure can rapidly alter glucose tolerance [106].

### 5.6. Exercise Training and Skeletal Muscle Satellite Cells

Adaptation of skeletal muscle to sustained mechanical challenge is controlled by SCs. SCs are quiescent cells located between the basal lamina and the sarcolemma and undergo rapid activation upon exposure to contraction‐induced mechanical stress [107]. Specifically, progressive resistance training regimes lasting 4–8 weeks induce a statistically significant expansion of the SC pool within human skeletal muscle compared to sedentary or nontraining control groups [108, 108]. Our result corroborates these findings, where the number of Ki‐67 immunoreactive SCs in the skeletal muscle of rats trained for 4–8 weeks is significantly higher than that of nontrained controls.

## 6. Conclusions

• After 2 weeks of regular moderate‐intensity physical activity, metabolic adjustment to exercise training becomes apparent: the levels of IL‐6 and IGF‐1 increase, and the expression of myostatin reduces. These changes positively modulate the level of blood glucose and decrease body weight.
• Physical training raises the mineralocorticoids‐to‐glucocorticoids ratio, and leptin reduces the levels of ghrelin. The effect becomes more pronounced with the time of training: if the experiment lasts over 4 weeks, physical exercises greatly affect hormonal regulation.
• After physical training, high levels of IL‐6 correlate with low secretion of ghrelin, which regulates appetite and impacts body weight. The high levels of IGF‐1 maintain electrolyte balance by raising aldosterone and reducing cortisol (Figures 10).
• Exercise training induces lasting metabolic improvements, including sustained reductions in blood glucose and an increase in body weight (attributable to muscle growth). Surprisingly, detraining periods maintained these benefits, implying durable muscle adaptations. This underscores the importance of sustained and consistent exercise for metabolic health.
• XGBoost and LightGBM models effectively predicted body weight and blood glucose, with exercise factors as key predictors. Total training duration was most important for weight management, while recent exercise had the greatest impact on blood glucose. These findings emphasize that long‐term exercise supports weight management, whereas regular recent activity is crucial for blood glucose regulation.
• PCA supplementation modulates the activity of glycolytic enzymes, changes the utilization of energy substrates, enhances aerobic capacity, and fulfills the metabolic resources of athletes. The supplement enhances the testosterone–cortisol ratio, further supporting metabolic reactions that adjust the individual to physical activity.

**FIGURE 10:**
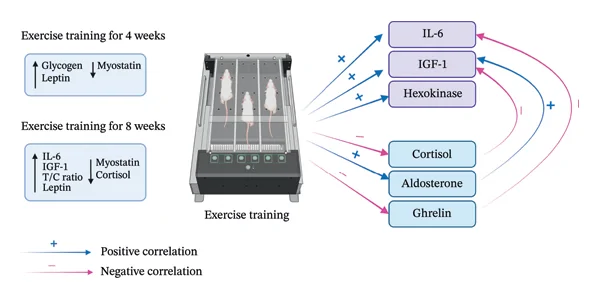
Summary diagram of the study results.

## 7. Study Strengths, Weaknesses, and Future Directions

The strengths of the study are as follows:

• One major strength of the study is the evaluation of the effects of different training settings on metabolic markers in an animal model. This approach allowed us to explore and identify an optimal exercise dose for improving metabolic health.
• The current study provides novel insights into the relationship between muscle‐secreted factors (myokines) and endocrine adaptations in response to exercise training as well as detraining.
• Study findings may inform translational research by helping to determine the optimal dose needed to achieve maximum metabolic and hormonal responses.

The study possesses the following limitations:

• We did not assess different detraining protocols, which could have offered more detailed insights into how quickly training adaptations are lost.
• The sample size was relatively small, potentially limiting the statistical power of the findings. Future studies should include larger animal cohorts, assess different detraining protocols, and perform more comprehensive hormonal and myokine analyses to enhance the translational relevance of the findings.
• Exercise in animal models is typically a conditioned behavior rather than a voluntary, intentional activity as seen in humans. This methodological difference limits the translational relevance of findings, as animal studies often reflect forced extremes of activity or inactivity rather than the natural spectrum of physical movement observed in human populations.
• The present study aimed to evaluate chronic exercise training adaptations and the cumulative effect rather than the acute effect of exercise. Blood and gastrocnemius muscle samples were collected 48 h post‐exercise to minimize the influence of transient physiological disturbances associated with the last exercise bout, including muscle damage, inflammatory markers, and acute metabolic changes [109, 110]. However, a single late time point may not fully identify the kinetics of exercise‐induced myokines, and endocrine adaptations occur before 48 h. Inclusion of shorter sampling time points would help to better distinguish acute from chronic adaptations and provide further insight into the temporal regulation of exercise‐induced myokines and hormonal responses.
• This study did not examine body composition, including fat mass and muscle mass. This limits the ability to determine whether exercise‐induced metabolic and biochemical adaptations are influenced by changes in fat and muscle mass.

Future directions:

• Future studies should be conducted using obesity and type 2 diabetes models to better understand the hormonal and metabolic adaptations. This will help to identify the dose and duration of exercise training to modulate endocrine and metabolic responses in disease states, as well as how long it takes to lose these adaptations when the body transitions from training to detraining. This will have significant clinical implications for designing exercise prescription and supplementation strategies in metabolic disease.
• Further studies should include multiple acute time points, such as 12 and 24 h post‐exercise, in addition to a chronic time point (48 h), for the collection of blood and muscle tissue. This will help to better characterize the impact of training on metabolism and to distinguish between the acute effect and cumulative training effects.

## Author Contributions

Conceptualization, Y. Statsenko, C. Platat, and H. Shakoor; methodology, Y. Statsenko and H. Shakoor; software, Y. Statsenko, A. Riabinin, and H. Shakoor; validation, Y. Statsenko and H. Shakoor; interpretation, Y. Statsenko, H. Shakoor, and E. Adeghate; formal analysis, H. Shakoor, Y. Statsenko, C. Platat, and E. Adeghate; investigation, H. Shakoor, J. Kizhakkayil, and C. Dsouza; data curation, H. Shakoor; resources, Y. Statsenko and C. Platat; writing–original draft preparation, H. Shakoor and Y. Statsenko; writing–review and editing, Y. Statsenko, C. Platat, H. Shakoor, E. Adeghate, and B. Sadek; animal work, H. Shakoor, J. Kizhakkayil, and A. Parveen; visualization, J. Kizhakkayil and A. Parveen; supervision, C. Platat and Y. Statsenko; project administration, Y. Statsenko and C. Platat; funding acquisition, Y. Statsenko.

## Funding

The study was supported by ASPIRE, the technology program management pillar of Abu Dhabi’s Advanced Technology Research Council (ATRC), via the ASPIRE Precision Medicine Research Institute Abu Dhabi (ASPIREPMRIAD), award grant number VRI‐20‐10. Portion of the study was supported by the Strategic Research Program of the Zayed Center for Health Sciences (grant number 12R379), the UAEU‐McGill Joint Research Project (grant number 12M286), and the CMHS faculty Research Grant (12M322).

## Disclosure

All authors have read and agreed to the published version of the manuscript.

## Ethics Statement

The animal study protocol was approved by the Animal Research Ethics Committee of the College of Medicine and Health Sciences at United Arab Emirates University (ERA_2021_7238, approved on 15 March 2021).

## Conflicts of Interest

The authors declare no conflicts of interest.

## Data Availability

The original contributions presented in this study are included in the article. Further inquiries can be directed to the corresponding author.
